# Bimetallic Thin-Walled Box Beam Thermal Buckling Response

**DOI:** 10.3390/ma15217537

**Published:** 2022-10-27

**Authors:** Sandra Kvaternik Simonetti, Goran Turkalj, Damjan Banić, Domagoj Lanc

**Affiliations:** Department of Engineering Mechanics, Faculty of Engineering, University of Rijeka, 51000 Rijeka, Croatia

**Keywords:** thin-walled, FEM, thermal buckling analysis

## Abstract

A beam model for thermal buckling analysis of a bimetallic box beam is presented. The Euler–Bernoulli–Vlasov beam theory is employed considering large rotations but small strains. The nonlinear stability analysis is performed using an updated Lagrangian formulation. In order to account for the thermal effects of temperature-dependent (TD) and temperature-independent (TID) materials, a uniform temperature rise through beam wall thickness is considered. The numerical results for thin-walled box beams are presented to investigate the effects of different boundary conditions, beam lengths and material thickness ratios on the critical buckling temperature and post-buckling responses. The effectiveness and accuracy of the proposed model are verified by means of comparison with a shell model. It is revealed that all of the abovementioned effects are invaluable for buckling analysis of thin-walled beams under thermal load. Moreover, it is shown that the TD solutions give lower values than the TID one, emphasizing the importance of TD materials in beams.

## 1. Introduction

Thin-walled beams and structures are increasingly used in engineering branches, in standalone forms and as a stiffeners for plate- and shell-like structures, due to their high strength and light weight. However, these structures show susceptibility to local buckling and buckling failure [1,2]. Buckling analysis and the post-buckling response of such weight-optimized structures have been the topic of many research papers, such as [3,4,5,6], especially in the field of composite materials [7,8,9,10,11].

If the thermal environment is considered, the stability of structures has received significant attention in recent years: Duan et al. [12] performed thermal analysis of a beam element, Saha and Ali [13] presented a post-buckling mathematical model of a slender road under uniform temperature rise, while Cui and Hu [14] analyzed the thermal buckling and vibration of a beam. Jeyaraj et al. [15] investigated experimental and theoretical non-uniform heating of an isotropic beam. Burgreen and Mannit [16] and Burgreen and Regal [17] analysed the thermal buckling of bimetallic beams. In the case of composite beams, Aydogdu [18] obtained critical buckling temperatures of composite beams, Luan et al. [19] presented an analytical solution for buckling and vibration of FG beams, Kiani and Eslami [20,21] investigated buckling analysis under different types of thermal loads, while Giunta [22] analyzed FG beams under thermal/mechanical load using the Carrera unified formulation. However, there are not many papers about thermal buckling analyses of thin-walled structures: Libresceu [23] studied stability problems in a high-temperature environment and Ziane et al. [24] studied analytical methods for buckling and vibration responses of porous beams under thermomechanical loads. 

In the present work, thermal buckling analysis of a thin-walled bimetallic box beam and frame structures is presented. The material is assumed to be linear, elastic and isotropic. The model is based on Euler–Bernoulli–Vlasov theory and on assumptions of large rotations and small strains. It is also assumed that the cross-section is not deformed in its own plane and that there are no shear strains in the middle surface. The nonlinear displacement field, which includes nonlinear displacement terms due to large rotation effects, is implemented. Using the UL description, the element geometric stiffness is derived. As an incremental iterative solution scheme, the Newton–Raphson method is used. Furthermore, this paper is a continuation of the research in which thermal buckling analysis of temperature-independent materials was conducted [25], which has now been further expanded with temperature-dependent materials’ properties. As far as the authors are aware, there is no beam model solution for thermal buckling analysis of thin-walled beam-type structures with temperature-dependent materials’ properties.

The numerical results for thin-walled box beams are presented to investigate the effects of different boundary conditions, namely clamped–clamped, simply supported and clamped–simply supported, beam lengths and material thickness ratios on the critical buckling temperature and post buckling responses. In order to demonstrate the accuracy of the numerical algorithm, benchmark examples using shell FEM code were developed. Numerical results show that the abovementioned effects have a huge impact on the buckling analysis.

## 2. Materials and Methods

### 2.1. Kinematics

Two sets of coordinate systems related to the angle of orientation β are considered. The first one is a Cartesian (*z, x, y*) coordinate system where the *z*-axis coincides with the longitudinal beam that passes through the centroid *O* of each cross section, while the *x*- and *y*-axes are principal axes. The second one is a contour coordinate system where the *s*-axis is tangential to the middle surface directed along the contour line of the cross-section while the *n*-axis is perpendicular to the s-axis.

The field of incremental displacement measures of a cross section are defined as [8]:

(1) $$\begin{array}{c} w_{0}=w_{0}\left(z\right);\;u_{s}=u_{s}\left(z\right);\;v_{s}=v_{s}\left(z\right);\;\varphi_{z}=\varphi_{z}\left(z\right);\\ \varphi_{x}=\varphi_{x}\left(z\right)=-\frac{dv_{s}}{dz};\varphi_{y}=\varphi_{y}\left(z\right)=\frac{du_{s}}{dz};\theta =\theta \left(z\right)=-\frac{d\varphi_{z}}{dz}\left(z\right), \end{array}$$

where $w_{0}$,$\;u_{s}$ and $v_{s}$ are the rigid-body translations of the cross-section centroid in the *z*-, *x*- and *y*-direction, respectively, while $\varphi_{z}$, $\varphi_{x}$ and $\varphi_{y}$ are the rigid-body rotations about the aforementioned axis; θ is a warping parameter of the cross-section.

In the case of small rotations, the incremental displacement field consists of the first-order displacement values:

(2) $$\begin{array}{c} u_{z}\left(z,x,y\right)=w_{0}\left(z\right)-y\frac{dv_{s}}{dz}\left(z\right)-x\frac{du_{s}}{dz}\left(z\right)-\omega \left(x,y\right)\frac{d\varphi_{z}}{dz}\left(z\right),\\ u_{x}\left(z,x,y\right)=u_{s}\left(z\right)-\left(y-y_{s}\right)\varphi_{z}\left(z\right)\\ u_{y}\left(z,x,y\right)=v_{s}\left(z\right)+\left(x-x_{s}\right)\varphi_{z}\left(z\right), \end{array}$$

where $u_{z}$, $u_{x}$ and $u_{y}$ are the linear displacement increments of an arbitrary point on the cross-section defined by the x and y coordinates and the warping function ω(x,y). When the large rotations are considered, nonlinear displacement increments are expressed as follows:

(3) $$\begin{array}{c} {\tilde{u}}_{z}\left(z,x,y\right)=0.5\left[-\left(x-x_{s}\right)\varphi_{z}\varphi_{x}+\left(y-y_{s}\right)\varphi_{z}\varphi_{y}\right],\\ {\tilde{u}}_{x}\left(z,x,y\right)=0.5\left\{-\varphi_{x}\varphi_{y}y-\left[\varphi_{z}^{2}+\varphi_{y}{}^{2}\right]x+x_{s}\varphi_{z}^{2}\right\},\\ {\tilde{u}}_{y}\left(z,x,y\right)=0.5\left\{-\varphi_{x}\varphi_{y}x-\left[\varphi_{z}^{2}+\varphi_{x}{}^{2}\right]y+y_{s}\varphi_{z}^{2}\right\},\; \end{array}$$

and should be added to those from Equation (2).

Considering the nonlinear displacement field, the Green–Lagrange strain tensor components can be written as:

(4) $$\begin{array}{c} \varepsilon_{ij}=\frac{1}{2}\left[{\left(u_{i}+{\tilde{u}}_{i}\right)}_{,j}+{\left(u_{j}+{\tilde{u}}_{j}\right)}_{,i}+{\left(u_{k}+{\tilde{u}}_{k}\right)}_{i}+{\left(u_{k}+{\tilde{u}}_{k}\right)}_{,j}\right]≅e_{ij}+\eta_{ij}+{\tilde{e}}_{ij},\\ 2e_{ij}=u_{i,j}+u_{j,i}\\ 2\eta_{ij}=u_{k,i}+u_{k,j}\\ 2{\tilde{e}}_{ij}={\tilde{u}}_{i,j}+{\tilde{u}}_{j,i}\; \end{array}$$

where $e_{ij}$ and $\eta_{ij}$ are the linear and nonlinear strain components corresponding to the linear displacement, while ${\tilde{e}}_{ij}$ is the linear strain component corresponding to the nonlinear displacement due to the large rotations.

The contour mid-line displacement $\bar{w}$, and $\bar{v}$ can be seen more detail in [26].

Due to the in-plane rigidity hypothesis of the cross-section, the non-zero strain components are [7]:

(5) $$e_{zz}=\frac{\partial w}{\partial z}\;,\;\;e_{zs}=\frac{\partial w}{\partial s}+\frac{\partial v}{\partial z}\;,\;$$

(6) $$\begin{array}{c} \eta_{zz}=\frac{1}{2}\left[{\left(\frac{\partial w}{\partial z}\right)}^{2}+{\left(\frac{\partial u}{\partial z}\right)}^{2}+{\left(\frac{\partial v}{\partial z}\right)}^{2}\right],\\ \eta_{zs}=\frac{\partial w}{\partial z}\frac{\partial w}{\partial s}+\frac{\partial u}{\partial z}\frac{\partial u}{\partial s}+\frac{\partial v}{\partial z}\frac{\partial v}{\partial s}, \end{array}$$

(7) $${\tilde{e}}_{zz}=\frac{\partial w}{\partial z}\;,\;\;{\tilde{e}}_{zs}=\frac{\partial w}{\partial s}+\frac{\partial v}{\partial z}.\;$$

The stress resultants of the beam can be defined as:

(8) $$\begin{array}{c} F_{z}=\overset{}{\underset{A}{\int }}\sigma_{z}dnds,\;\\ M_{x}=\overset{}{\underset{A}{\int }}\sigma_{z}\left(y-n\cos\beta \right)dnds,\;\;M_{y}=\overset{}{\underset{A}{\int }}\sigma_{z}\left(x+n\sin\beta \right)dnds,\;\;\\ M_{t}=\overset{}{\underset{A}{\int }}\tau_{sz}\left(n+\frac{F_{s}}{t}\right)dnds,\;\;M_{\omega }=\overset{}{\underset{A}{\int }}\sigma_{z}\left(\omega -nq\right)dnds, \end{array}$$

where $F_{z}$ represents axial force, $M_{x}$ and $M_{y}$ are bending moments with respect to *x-* and *y-*axis, respectively, $M_{t}$ is the torsion moment and $M_{\omega }$ is the warping moment (bimoment). t is the thickness of the closed section contour and $F_{s}$ is the St. Venant circuit flow [26]. 

### 2.2. Constitutive Equations

**Figure 1:**
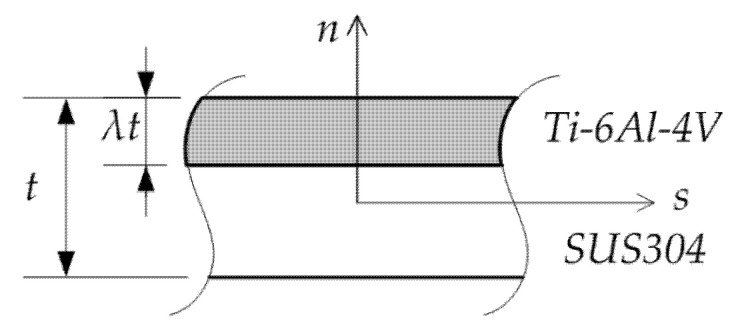
Bimetallic beam wall.

Consider a bimetallic beam made of two different metals. A beam wall with a core thickness of Ti–6Al–4V λt on the outer surface and SUS304 in the inner part of cross-section beam wall is shown in Figure 1. It is assumed that the layers of materials are perfectly bonded. 

If the thermo-elastic material properties are considered as a function of temperature *T*, they can be calculated for each material, as described in [19,27]:

(9) $$P\left(T\right)=P_{0}\left(1+P_{-1}T^{-1}+P_{1}T+P_{2}T^{2}+P_{3}T^{3}\right),$$

where *P* represents Young’s modulus E and thermal expansion coefficient α, while $P_{0}$, $P_{-1}$,$\;P_{1}$, $P_{2}$ and $P_{3}$ are temperature-dependent coefficients listed in Table 1 for different metals [28]. For simplicity, Poisson’s ratio ν is assumed to be constant, ν=0.3.

It is assumed that the temperature of the whole beam is uniform and increased from the current ambient temperature $T_{0}$ to the critical value in incremental steps of 1 °C. If the axial displacements are prevented, the temperature at a point T(n,z) may be raised to T+ΔT, in the way that the beam buckles. ΔT is the temperature rise. The temperature that is read as the critical buckling temperature is the temperature difference compared to the ambient temperature. The process can be described as quasi-adiabatic since the heat exchange between the environment and the beam is neglected.

The stress–strain relations of the bimetallic beam can be written as:

(10) $$\left(\begin{array}{c} \sigma_{z}\\ \tau_{sz} \end{array}\right)=\left(\begin{array}{cc} E\left(n,z,T\right) & 0\\ 0 & G\left(n,z,T\right) \end{array}\right)\cdot \left(\begin{array}{c} e_{zz}-\alpha \left(n,z,T\right)\Delta T\\ \gamma_{sz} \end{array}\right),$$

where

(11) $$G\left(n,z\right)=\frac{E\left(n,z,T\right)}{2\left[1+\nu \left(n,z,T\right)\right]}.$$

Using Equations (5), (10) and (11), the beam forces can be expressed in a matrix form as:

(12) $$\left\{\begin{array}{c} F_{z}\\ M_{y}\\ M_{x}\\ M_{\omega }\\ M_{t} \end{array}\right\}=\left[\begin{array}{ccccc} R_{11} & R_{12} & R_{13} & R_{14} & 0\\ R_{21} & R_{22} & R_{23} & R_{24} & 0\\ R_{31} & R_{32} & R_{33} & R_{34} & 0\\ R_{41} & R_{42} & R_{43} & R_{44} & 0\\ 0 & 0 & 0 & 0 & R_{55} \end{array}\right]\left\{\begin{array}{c} dw_{0}/dz\\ -d^{2}u_{s}/dz^{2}\\ -d^{2}v_{s}/dz^{2}\\ -d^{2}\varphi_{z}/dz^{2}\\ 2d\varphi_{z}/dz \end{array}\right\}-\left\{\begin{array}{c} N_{z}^{T}\\ M_{y}^{T}\\ M_{x}^{T}\\ M_{\omega }^{T}\\ 0 \end{array}\right\},$$

where $R_{ij}$ represents the thin-walled beam stiffness, as shown in Appendix A. $N_{z}^{T},\;M_{x}^{T},\;M_{y}^{T}$ and $M_{\omega }^{T}$ are thermal force and thermal moments, respectively:

(13) $$\begin{array}{c} N_{z}^{T}=\overset{}{\underset{A}{\int }}E\left(n,z,T\right)\alpha \left(n,z,T\right)\Delta Tdnds;\\ M_{x}^{T}=\overset{}{\underset{A}{\int }}E\left(n,z,T\right)\alpha \left(n,z,T\right)\Delta T\left(y-n\cos\beta \right)dnds\\ M_{y}^{T}=\overset{}{\underset{A}{\int }}E\left(n,z,T\right)\alpha \left(n,z,T\right)\Delta T\left(x+n\sin\beta \right)dnds\\ M_{\omega }^{T}=\overset{}{\underset{A}{\int }}E\left(n,z,T\right)\alpha \left(n,z,T\right)\Delta T\left(\omega -nq\right)dnds \end{array}$$

### 2.3. Finite Element Formulation

**Figure 2:**
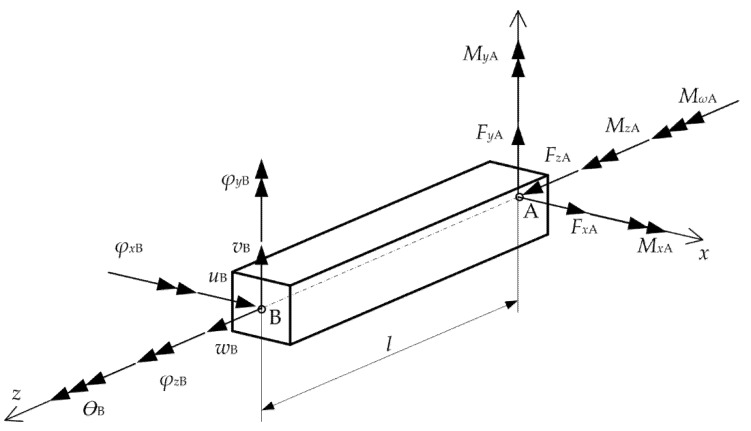
Nodal force vectors and displacements.

A two-nodded beam element with 14 degrees of freedom is shown in Figure 2. The nodal displacements and nodal force vectors are as follows:

(14) $${\left(u^{e}\right)}^{T}=\left\{w_{A}\;u_{A}\;v_{A}\;\varphi_{zA}\;\varphi_{xA}\;\varphi_{yA}\;w_{B}\;u_{B}\;v_{B}\;\varphi_{zB}\;\varphi_{xB}\;\varphi_{yB}\;\theta_{A}\;\theta_{B}\right\}\;$$

(15) $${\left(f^{e}\right)}^{T}=\left\{F_{zA}\;F_{xA}\;F_{yA}\;M_{zA}\;M_{xA}\;M_{yA}\;F_{zB}\;F_{xB}\;F_{yB}\;M_{zB}\;M_{xB}\;M_{yB}\;M_{\omega A}\;M_{\omega B}\right\}$$

where the superscript *e* denotes the *e*th finite element. It should be noted that nodal displacement w and nodal forces $F_{z},\;M_{x}$ and $M_{y}$ are defined in the centroid O, while other nodal components are defined in the shear center.

Applying the principle of the virtual work, the incremental equilibrium equations of a beam element in linearized form are:

(16) $$\delta U_{E}+\delta U_{G}=\delta^{2}W-\delta^{1}W,\;$$

where the equations from the left side consist of incremental virtual elastic strain energy:

(17) δUE=∫1VC1ijkl 1 ekl δ1 eij1 dV, 

and the incremental virtual geometric potential:

(18) δUG=∫1VS1ij δ1 ηij1 dV+∫VS1ij δ1 e˜ij1 dV−∫A1σt1i δu˜i1 dAσ. 

On the right side of the equations, the terms represent the virtual work completed by external forces at the end and at the beginning of the present increment:

(19) δ2W=∫A1σt2i δui1 dAσ,δ1W=∫V1S1ij δ1 eij1 dV=∫A1σt1i δui1 dAσ. 

In these equations, $S_{ij}$ is the second Piola–Kirchoff stress tensor, $t_{i}$ denotes the surface tractions, $C_{ijkl}$ presents the stress–strain tensor and the symbol δ indicates virtual quantities. By applying the linear interpolation functions for $w_{0}$ displacement and cubic interpolations for $w_{s}$, $u_{s}$ and $v_{s}$, one can obtain:

(20) $$\delta U_{E}=\overset{}{\underset{V}{\int }}S_{\mathrm{ij}}\delta e_{\mathrm{ij}}dV={\left(\delta u^{e}\right)}^{T}k_{E}^{e}u^{e},\;$$

(21) $$\delta U_{G}=\overset{}{\underset{V}{\int }}S_{\mathrm{ij}}\left(\delta \eta_{\mathrm{ij}}+\delta {\tilde{e}}_{\mathrm{ij}}\right)dV-\overset{}{\underset{A_{\sigma }}{\int }}t_{i}\delta {\tilde{u}}_{i}dA_{\sigma }={\left(\delta u^{e}\right)}^{T}k_{G}^{e}u^{e}$$

(22) $$\delta W=\overset{}{\underset{A_{\sigma }}{\int }}t_{i}\delta u_{i}dA_{\sigma }={\left(\delta u^{e}\right)}^{T}f^{e}\;$$

where $f^{e}$ is the nodal force vector, $k_{E}^{e}$ is the elastic stiffness matrix and $k_{G}^{e}$ is the geometric stiffness matrix of the beam element. Nonlinear equilibrium equations are solved using the Newton–Raphson method as incremental iterative approach [29,30], and the explicit form of the terms given in nonlinear components were described previously in [31]. 

## 3. Results and Discussion

**Figure 3:**
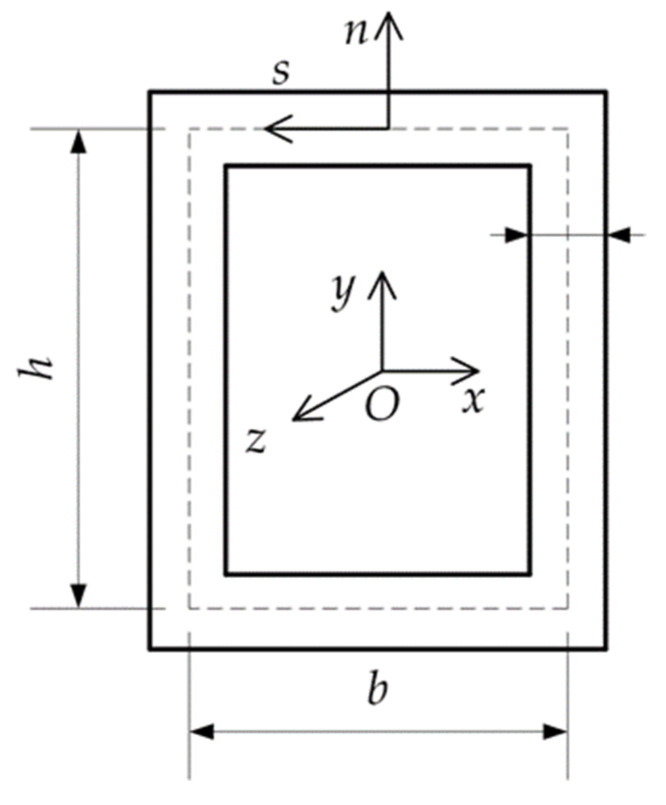
Box beam cross-section.

In numerical examples, the thin-walled box beam with height h=100 mm, width b=150 mm and thickness t=10 mm is considered (Figure 3). For verification purposes, the critical buckling temperatures were obtained by shell FEM commercial code [32]. In order to simulate the bimetallic material, the beam walls were divided into two layers of different metals with a variable thickness ratio *λ.* Note that for λ=0, the beam wall is fully SUS304, while as the index thickness ratio *λ* increases, the beam wall becomes fully Ti-6Al-4V.

### 3.1. Box Beam

In the first example, the eigenvalue results of the box beam for different boundary conditions, which are clamped–clamped (C-C), clamped–simply supported (C-S) and simply supported (S-S), beam lengths of $L_{1}=6\;m$, $L_{2}=8\;m$ and $L_{3}=10\;m$ and different material thickness ratios *λ* are given in Table 2, Table 3 and Table 4. Critical buckling temperatures are given for the first two flexural buckling modes. As the thickness ratio *λ* increases, the critical buckling temperatures increase as well due to the material properties of TI-6Al-4V. Furthermore, longer beams obtain lower critical buckling temperatures. As expected, clamped–clamped beams exhibit the highest eigenvalues for every beam length. Good agreement of the present results and solutions derived from the 2D model for both flexural buckling modes is achieved.

**Figure 4:**
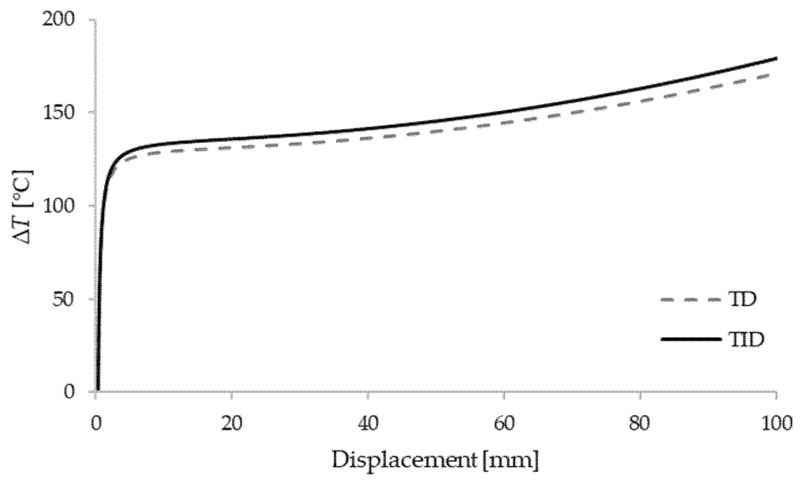
Critical temperatures vs. displacement for a clamped–clamped beam $L_{1}=6\;m$ with λ=0.2.

**Figure 5:**
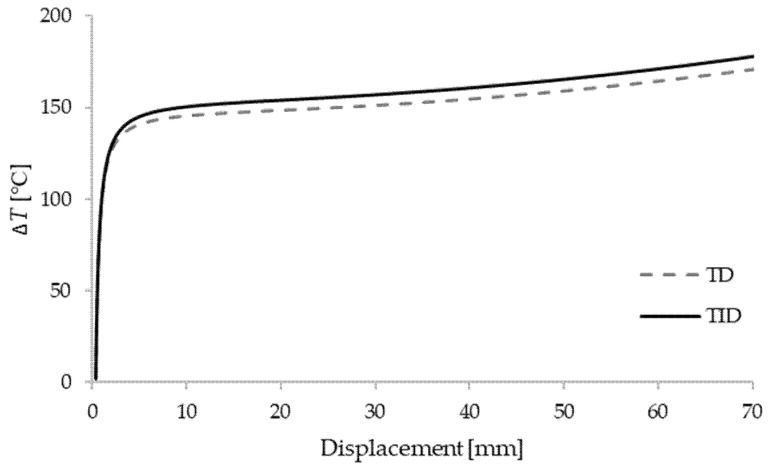
Critical temperatures vs. displacement for a clamped–clamped beam $L_{1}=6\;m$ with λ=0.5.

**Figure 6:**
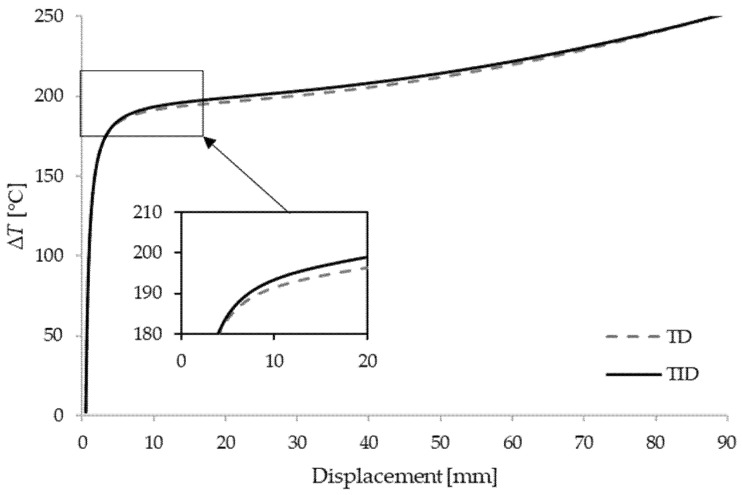
Critical temperatures vs. displacement for a clamped–clamped beam $L_{1}=6\;m$ with λ=0.8.

In order to further perform the nonlinear stability analysis of the box beam, a perturbation force of intensity ΔF=500 N is introduced in the *y*-axis direction at the midpoint of the clamped–clamped beam. Temperature–displacement curves of the shortest beam with the comparison of temperature-dependent (TD) and temperature-independent (TID) materials are shown in Figure 4, Figure 5 and Figure 6. The results are given for different thickness ratios: λ=0.2, λ=0.5 and λ=0.8. It can be seen that curves match very well with the critical buckling temperatures achieved in the eigenvalue manner. As expected, TD solutions obtained lower critical buckling temperatures. In the case of λ=0.2, the difference in critical temperature is around 4%, for λ=0.5 the difference is 3.3%, and for λ=0.8 it is 1.5%.

### 3.2. L-Frame

**Figure 7:**
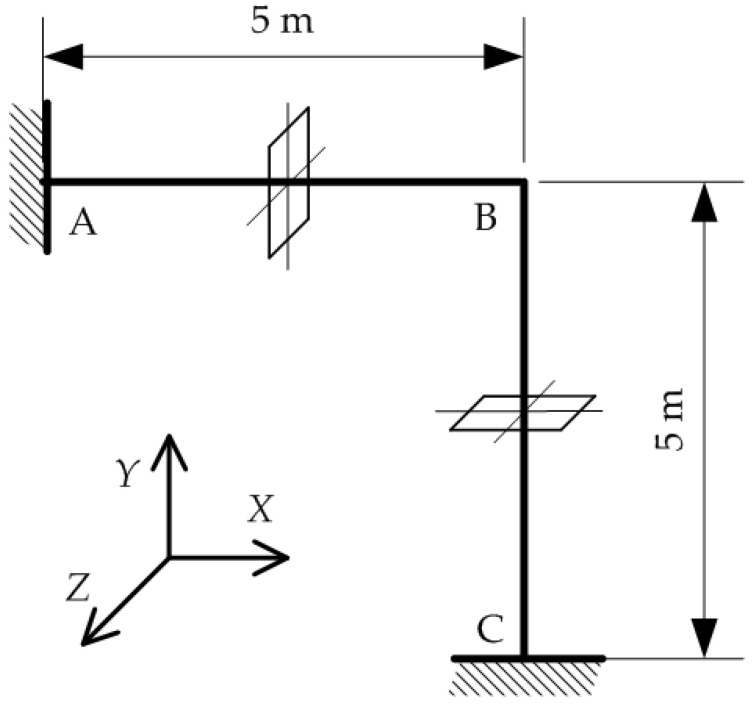
L-frame.

Furthermore, the model is tested for thermal buckling analysis of an L-frame with the length of both legs being L=5 m and cross-section described in the previous chapter (Figure 7). The frame is fixed at points A and C, while at point B, in-plane translations are prevented. 

**Figure 8:**
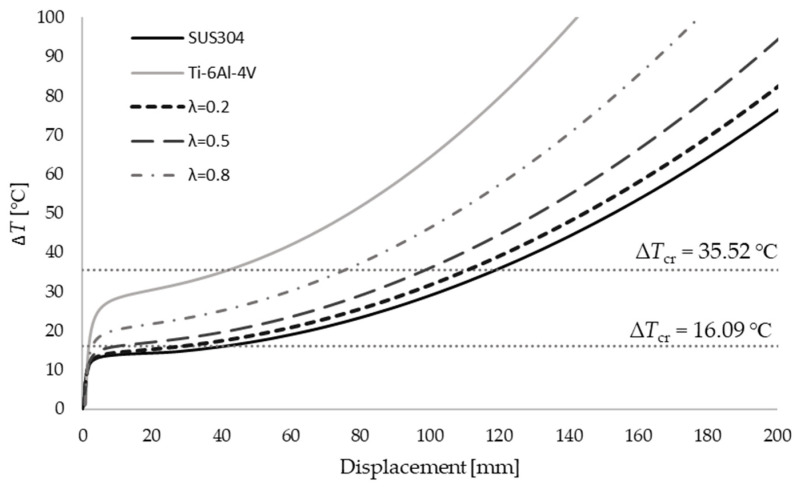
Displacements of the L-frame for several values of the material thickness ratio.

To verify the results, the critical buckling temperatures for the full SUS304,$\;\Delta T_{cr\left(SUS304\right)}=16.09\;℃$, and full Ti-6Al-4V $\Delta T_{cr\left(\mathrm{Ti}-6\mathrm{Al}-4V\right)}=35.52\;℃$, sections are solved by a shell commercial code. To perform nonlinear analysis, a small perturbation force ΔF=50 N acting in the *z*-axis direction at point *B* is applied. The results are shown in Figure 8 for pure metals and for λ=0.2t, λ=0.5t and λ=0.8t. The good recognition of the critical values can be noted. It can be observed that with an increase in the proportion of Ti-6Al-4V material, higher critical temperatures are achieved.

## 4. Conclusions

A thin-walled beam model capable of thermal buckling analysis has been presented. By means of the updated Lagrangian formulation, the incremental equilibrium equations have been developed using the nonlinear displacement field of the cross-section, taking into account the effects of large rotations. The reliability of the present model was verified by studying the benchmark examples, and the values obtained with the proposed model are in good agreement with those of the shell model. The effects of boundary conditions, the length of the beam and material thickness ratio on the critical buckling temperature and post-buckling response are of great importance. Additionally, it is shown that the TD solutions provide lower values than those of the TID solutions. The model was found to be efficient in predicting eigenvalues and nonlinear buckling behavior.

## Figures and Tables

**Table 1:** Temperature dependent coefficients [19].

Material	Properties	P_0_	P_1_	P_1_	P_2_	P_3_
Ti–6Al–4V	E (Pa)	122.56 × 10^9^	0.0	−4.586 × 10^−4^	0.0	0.0
	α (1/K)	7.5788 × 10^−6^	0.0	6.638 × 10^−4^	–3.147 × 10^−6^	0.0
SUS304	E (Pa)	201.04 × 10^9^	0.0	3.079 × 10^−4^	–6.534 × 10^−7^	0.0
	α (1/K)	12.330 × 10^−6^	0.0	8.086 × 10^−4^	0.0	0.0

**Table 2:** Critical buckling temperatures of the beam $L_{1}=6\;m$ for different boundary conditions and material thickness ratios.

			λ						
BC	Mode	Method	0	0.2	0.4	0.5	0.6	0.8	1
C-C	*Y*	Present	128.87	135.26	145.83	153.64	164.09	199.19	284.51
		Shell	130.22	138.77	151.18	159.72	170.71	205.83	287.97
	*X*	Present	239.24	252.56	273.89	288.9	308.62	373.43	528.16
		Shell	238.95	254.69	277.45	293.11	313.24	377.6	527.52
C-S	*Y*	Present	66.58	69.91	75.39	79.44	84.84	102.97	146.98
		Shell	65.98	70.313	76.604	80.936	86.504	104.29	145.66
	*X*	Present	122.87	130.02	140.09	148.58	158.73	192.03	271.48
		Shell	120.46	128.39	139.88	147.78	157.93	190.37	265.92
S-S	*Y*	Present	32.72	34.368	37.07	39.06	41.72	50.62	72.24
		Shell	32.196	34.314	37.39	39.507	42.226	50.904	71.077
	*X*	Present	60.28	60.29	69.07	72.86	77.83	94.15	133.08
		Shell	58.725	62.6	68.208	72.063	77.015	92.827	129.64

**Table 3:** Critical buckling temperatures of the beam $L_{2}=8\;m$ for different boundary conditions and material thickness ratios.

			λ						
BC	Mode	Method	0	0.2	0.4	0.5	0.6	0.8	1
C-C	*Y*	Present	73.17	76.83	82.85	87.3	93.24	113.16	161.54
		Shell	73.542	78.368	85.375	90.2	96.405	116.24	162.35
	*X*	Present	135.52	142.96	154.88	163.38	174.53	211.15	298.53
		Shell	135.02	143.91	156.78	165.62	177	213.37	298.08
C-S	*Y*	Present	37.63	39.52	42.63	44.91	47.97	58.21	83.07
		Shell	37.323	39.774	43.33	45.783	48.933	58.997	82.396
	*X*	Present	69.35	73.33	79.45	83.81	89.53	108.31	153.09
		Shell	68.256	72.754	79.261	83.736	89.487	107.87	150.68
S-S	*Y*	Present	18.45	19.38	20.9	22.03	23.53	28.55	40.73
		Shell	18.211	19.409	21.149	22.346	23.885	28.793	40.204
	*X*	Present	33.95	35.9	38.9	41.04	43.84	53.03	74.95
		Shell	33.278	35.473	38.651	40.836	43.642	52.603	73.466

**Table 4:** Critical buckling temperatures of the beam $L_{3}=10\;m$ for different boundary conditions and material thickness ratios.

			λ						
BC	Mode	Method	0	0.2	0.4	0.5	0.6	0.8	1
C-C	*Y*	Present	47.03	49.39	53.27	56.13	59.95	72.75	103.83
		Shell	47.158	50.252	54.745	57.839	61.818	74.536	104.11
	*X*	Present	86.74	91.72	99.37	104.82	111.98	135.47	191.49
		Shell	86.605	92.308	100.56	106.23	113.53	136.86	191.19
C-S	*Y*	Present	24.14	25.35	27.35	28.81	30.77	37.34	53.28
		Shell	23.965	25.538	27.823	29.397	31.419	37.881	52.906
	*X*	Present	44.43	46.99	50.91	53.71	57.38	69.4	98.09
		Shell	43.872	46.763	50.945	53.822	57.518	69.335	96.854
S-S	*Y*	Present	11.82	12.42	13.39	14.11	15.07	18.29	26.09
		Shell	11.695	12.464	13.582	14.35	15.338	18.491	25.818
	*X*	Present	21.74	22.99	24.91	26.28	28.08	33.96	47.99
		Shell	21.396	22.807	24.85	26.255	28.059	33.82	47.234

## Data Availability

Not applicable.

## Appendix A

$$\begin{array}{c} R_{11}=\overset{}{\underset{A}{\int }}E\left(n,z,T\right)dnds,\\ R_{12}=R_{21}=\overset{}{\underset{A}{\int }}E\left(n,z,T\right)\left(x+n\sin\beta \right)dnds,\\ R_{13}=R_{31}=\overset{}{\underset{A}{\int }}E\left(n,z,T\right)\left(y-n\cos\beta \right)dnds,\\ R_{14}=R_{41}=\overset{}{\underset{A}{\int }}E\left(n,z,T\right)\left(\omega -nq\right)dnds,\\ R_{22}=\overset{}{\underset{A}{\int }}E\left(n,z,T\right){\left(x+n\sin\beta \right)}^{2}dnds,\\ R_{23}=R_{32}=\overset{}{\underset{A}{\int }}E\left(n,z,T\right)\left(x+n\sin\beta \right)\left(y-n\cos\beta \right)dnds,\\ R_{23}=R_{32}=\overset{}{\underset{A}{\int }}E\left(n,z,T\right)\left(x+n\sin\beta \right)\left(y-n\cos\beta \right)dnds,\\ R_{24}=R_{42}=\overset{}{\underset{A}{\int }}E\left(n,z,T\right)\left(x+n\sin\beta \right)\left(\omega -nq\right)dnds,\\ R_{33}=\overset{}{\underset{A}{\int }}E\left(n,z,T\right){\left(y-n\cos\beta \right)}^{2}dnds,\\ R_{34}=R_{43}=\overset{}{\underset{A}{\int }}E\left(n,z,T\right)\left(y-n\cos\beta \right)\left(\omega -nq\right)dnds,\\ R_{44}=\overset{}{\underset{A}{\int }}E\left(n,z,T\right){\left(\omega -nq\right)}^{2}dnds,\\ R_{55}=\overset{}{\underset{A}{\int }}G\left(n,z,T\right){\left(n+\frac{F_{s}}{t}\right)}^{2}dnds. \end{array}$$
